# Dyslipidemia and metabolic syndrome in childhood-onset systemic lupus erythematosus: is it time to screen?

**DOI:** 10.1186/s12944-024-02395-4

**Published:** 2024-12-18

**Authors:** Sirin Nuntasri, Sirirat Charuvanij, Kraisoon Lomjansook, Puthita Saengpanit, Kwanjai Chotipanang, Maynart Sukharomana

**Affiliations:** 1 Division of Rheumatology, Department of Pediatrics, Faculty of Medicine Siriraj Hospital, Mahidol University, 2 Wanglang Road, Bangkoknoi, Bangkok, 10700 Thailand; 2 Division of Nephrology, Department of Pediatrics, Faculty of Medicine Siriraj Hospital, Mahidol University, Bangkok, Thailand; 3 Division of Nutrition, Department of Pediatrics, Faculty of Medicine Siriraj Hospital, Mahidol University, Bangkok, Thailand

**Keywords:** Systemic lupus erythematosus, Dyslipidemia, Hyperuricemia, Metabolic syndrome, Child, Adolescent, Childhood-onset

## Abstract

**Background:**

Childhood-onset systemic lupus erythematosus (cSLE) is associated with significant morbidity and mortality. Dyslipidemia and metabolic syndrome are recognized risk factors for premature atherosclerosis. This study aimed to determine the prevalence of dyslipidemia and metabolic syndrome, and to explore the relationships between lipid profiles, anthropometry, and disease status in cSLE.

**Methods:**

This cross-sectional study was conducted at a university-based tertiary referral center from April 2023-March 2024. Patients aged 10–19 years with cSLE diagnosed before 18 years and at least 1 year follow-up were enrolled, excluding those with other autoimmune disorders, chronic kidney disease, infections, receiving lipid lowering drugs prior, and pregnancy. Demographic data, metabolic laboratory tests, disease status, dietary intake, anthropometry, and body composition via bioelectric impedance analysis were evaluated. The prevalence of dyslipidemia and metabolic syndrome were documented. Variables were compared between patients with and without dyslipidemia. Correlations between lipid profiles, metabolic laboratory variables, and SLE disease-related variables were explored.

**Results:**

A total of 132 cSLE patients (94.7% female, mean age 11.6 ± 2.6 years) were included. Dyslipidemia was present in 48.5%, hypertriglyceridemia being the most common (28.8%); metabolic syndrome and hyperuricemia were present in 3.8% and 20.5%, respectively. Patients with dyslipidemia were significantly younger at cSLE diagnosis, had higher percentage of hypertension and active features of organ involvement, lower percentage of Lupus Low Disease Activity State, more use of mycophenolate mofetil and antihypertensive medications, higher uric acid level, higher waist circumference, body mass index, body mass index z-score, and fat mass (*P* < 0.05). Triglycerides, low-density lipoprotein cholesterol, and total cholesterol correlated positively with urine protein-to-creatinine ratio (*r* = 0.472, 0.469, and 0.591, respectively; *P* < 0.001) and negatively with serum albumin (*r* = -0.372, -0.506, and − 0.528, respectively; *P* < 0.001). Total cholesterol and low-density lipoprotein cholesterol correlated positively with cumulative prednisolone equivalent dose (rho = 0.350 and rho = 0.351, respectively, *P* < 0.001).

**Conclusions:**

Nearly half of cSLE patients had dyslipidemia, especially those with younger age at diagnosis, higher body mass index, proteinuria, and suboptimal-controlled disease. Metabolic syndrome and hyperuricemia were present. Lipid profile assessment in early adolescents is recommended to identify metabolic comorbidities in cSLE.

## Background

Systemic lupus erythematosus (SLE) is a chronic autoimmune disease affecting multiple organs and tissues, leading to significant morbidity and mortality [1]. In childhood-onset SLE (cSLE), there is increased risk for cardiovascular disease-related mortality compared to age-matched healthy children and young people [2, 3]. Subclinical atherosclerosis has been reported up to 32% of cSLE patients [4]. Moreover, subclinical atherosclerosis and increased carotid intima-media thickness have been observed in cSLE, especially during disease flares [5]. Recently, distinct subclinical atherosclerosis progression trajectories were identified in cSLE [3].

Dyslipidemia is a traditional risk factor for cardiovascular disease, although its pathogenesis in SLE remains multifactorial and poorly understood [6, 7]. Dyslipidemia has gained attention in SLE patients due to higher incidence of cardiovascular and cerebrovascular diseases compared to the general population [8–12], contributing to significant morbidity and mortality [13–16]. Metabolic syndrome, commonly associated with dyslipidemia, is another important comorbidity in SLE. Multiple criteria, such as those from the International Diabetes Federation [17] and Third Report of the National Cholesterol Education Program Expert Panel on Detection, Evaluation, and Treatment of High Blood Cholesterol in Adults (NCEP-ATP III) [18], have been applied to define metabolic syndrome in children. A previous study linked metabolic syndrome in cSLE patients with younger age [19]. Given the increasing cardiovascular risks in SLE, routine assessment of dyslipidemia and metabolic syndrome is critical. The International Consensus for Provisions of Quality-Driven Care in cSLE has recommended education about cardiovascular risk factors for patients age 13 years or older and parents, with regular assessment of lipid profile levels every 2 years [20]. However, the knowledge gap appears in cSLE patients who may potentially be at risk for these comorbidities at the age younger than the standard recommendation for assessment. Moreover, studies focusing on dyslipidemia, metabolic syndrome, and the associations to cSLE disease are limited, particularly in Southeast Asian populations.

This study aimed to determine the prevalence of dyslipidemia and metabolic syndrome in cSLE, and to explore the relationships between lipid profiles, anthropometry, and disease status.

## Methods

This cross-sectional study was performed prospectively in cSLE patients aged 10–19 years at the Department of Pediatrics, Faculty of Medicine Siriraj Hospital, Mahidol University, Thailand. The setting was a single-center, university-based tertiary referral hospital. The cSLE patients were recruited at the outpatient clinics of the Division of Rheumatology and the Division of Nephrology, both of which provided continuous care for cSLE patients. The research team members and a research associate were involved in the recruitment process, with facilitation by nurses of outpatient clinics. Participants were enrolled and assessed for data collection once, cross-sectionally, between April 2023-March 2024.

Participants were cSLE patients diagnosed before 18 years of age. Their diagnoses were based on the classification criteria of the American College of Rheumatology (ACR) 1997 [21], Systemic Lupus International Collaborating Clinics (SLICC) 2012 [22], or the European League Against Rheumatism/ACR 2019 criteria [23]. All patients had a disease duration and follow-up period of at least 12 months. In this study, the rationale for selecting patients age 10–19 years were the following: the lower limit at age 10 years was selected to correspond with the definition of metabolic syndrome by the International Diabetes Federation criteria according to age group which has been clearly established for age 10 years and older [17], and the upper limit at age 19 years was selected to correspond with the study inclusion criteria of cSLE diagnosis before age 18 years with one year of disease duration and follow-up, and therefore could potentially have patients age 19 years at the time of assessment. In the context of our study, we did not include cSLE patients below age 10 years as there was no definition for metabolic syndrome for this age group, but a definition for at-risk group in ages 6 to below 10 years [17]. The exclusion criteria were patients who declined to participate, could not communicate in Thai, were on or had history of receiving lipid lowering drugs to prior to enrollment, had chronic kidney disease, had infection, had other autoimmune disorders, were pregnant, or had limitations preventing body composition assessment via bioelectric impedance analysis.

The study was conducted in accordance with the principles of the Declaration of Helsinki. Approval was obtained from the Siriraj Institutional Review Board, Faculty of Medicine Siriraj Hospital, Mahidol University (certificate of approval Si 190/2023). Written informed consent and assent were obtained from all participants.

Demographic data, cumulative organ involvements until study enrollment, current treatments, and medication dosages including daily prednisolone and cumulative prednisolone equivalent doses were collected from medical records. Comorbidities and family history of metabolic conditions—including hypertension, diabetes mellitus, dyslipidemia, and metabolic syndrome—were assessed. Dietary intake was evaluated using a 3-day food record, documenting food consumption over 3 consecutive days before the assessment date [24]. Macronutrient and caloric intake were analyzed by dietitians using the Institute of Nutrition, Mahidol University Calculation (INMUCAL) nutritional analysis program, version 4.0 [25].

On the scheduled assessment date, anthropometric weight, height, and waist circumference measurements were obtained. Weight-for-age, height-for-age, weight-for-height percentages, and body mass index (BMI) z-scores were calculated using the World Health Organization growth references for ages 5–19 years and WHO AnthroPlus software [26, 27]. Body composition was assessed in the supine position using multi-frequency bioelectric impedance analysis (InBody S10; Inbody Co Ltd, Seoul, Korea) [28], which can provide more accurate and detailed assessment of total body water and body composition.

After a 12-hour fast, blood samples were collected for various analyses. These analyses included lipid profiles, glucose metabolism markers, uric acid, immunologic testing to evaluate disease activity of cSLE, hematology tests, with renal and liver function tests. The lipid profiles were total cholesterol (TC), triglycerides (TG), high-density lipoprotein cholesterol (HDL-C), and direct low-density lipoprotein cholesterol (LDL-C). The glucose metabolism markers were fasting blood glucose (FBG) and hemoglobin A1c (HbA1c). Immunologic testing included complement levels (C3 and C4), antinuclear antibodies, and anti-double-stranded DNA antibodies. The assessment methods for the laboratory analysis were as follows: TC: enzymatic colorimetric method; TG: enzymatic colorimetric method; HDL-C: homogenous enzymatic method; LDL-C: homogenous enzymatic colorimetric assay; FBG: enzymatic (hexokinase) method; HbA1c: immunoassay and high performance lipid chromatography; uric acid: enzymatic colorimetric method; antinuclear antibodies: indirect immunofluorescence assay and chemiluminescence assay; anti-double-stranded DNA antibodies: indirect immunofluorescence assay and enzyme linked immunosorbent assay; C3 and C4: nephelometry. Hematology tests were complete blood count and erythrocyte sedimentation rate (ESR). Urinalysis and first-morning urine protein-to-creatinine ratio (UPCR) were obtained.

The SLE disease activity was evaluated using the Systemic Lupus Erythematosus Disease Activity Index 2000 (SLEDAI-2 K) [29] and the Lupus Low Disease Activity State (LLDAS) criteria, defined by a SLEDAI-2 K score of less than 4 without major organ activity, a Physician Global Assessment score below 1 (scale 0–3), a daily prednisone dose of 7.5 mg or less, and stable immunosuppressive therapy [30]. Disease damage was assessed using the SLICC/ACR damage index [31].

Lipid profiles were stratified according to the National Cholesterol Education Program Expert Panel on Cholesterol Levels in Children [32]. For TC, levels were classified as acceptable (< 170 mg/dL), borderline high (170–199 mg/dL), and high (≥ 200 mg/dL). For LDL-C, levels were acceptable (< 110 mg/dL), borderline high (120–129 mg/dL), and high (≥ 130 mg/dL). For TG in ages 10–19 years, levels were acceptable (< 90 mg/dL), borderline high (90–129 mg/dL), and high (≥ 130 mg/dL). For HDL-C, levels were acceptable (> 45 mg/dL), borderline low (40–45 mg/dL), and low (< 40 mg/dL).

In this study, “dyslipidemia” was defined as the presence of at least one abnormal lipid profile as follows: high TC (≥ 200 mg/dL), high LDL-C (≥ 130 mg/dL), high TG (≥ 130 mg/dL), or low HDL-C (< 40 mg/dL) [32]. “Metabolic syndrome” was defined according to International Diabetes Federation criteria, which diagnose patients aged at least 10 years; the definition of metabolic syndrome in children and adolescents age 10 to less than 16 years were obesity ≥ 90th percentile (or adult cutoff if lower) as assessed by waist circumference, plus any two of the following: TG ≥ 150 mg/dL (1.7 mmol/L), HDL-C < 40 mg/dL (1.03 mmol/L), systolic blood pressure ≥ 130 mmHg or diastolic blood pressure ≥ 85 mmHg, FBG ≥ 100 mg/dL (5.6 mmol/L) or known type 2 diabetes mellitus [33]; for age 16 years or older, the adult definition for metabolic syndrome definition were applied consisting of waist circumference ≥ 90 cm for men or ≥ 80 cm for women, plus any two of the following: raised TG ≥ 150 mg/dL (1.7 mmol/L) or specific treatment for this lipid abnormality, reduced HDL-C < 40 mg/dl (1.03 mmol/L) in men or < 50 mg/L (1.29 mmol/L) in women or specific treatment for this lipid abnormality, raised systolic blood pressure ≥ 130 mmHg or diastolic blood pressure ≥ 85 mmHg or treatment of previously diagnosed hypertension, raised FBG ≥ 100 mg/dL (5.6 mmol/L) or previously diagnosed type 2 diabetes mellitus [17, 33].

“Hyperuricemia” was defined based on the Canadian Laboratory Initiative on Pediatric Reference Intervals [34]; for individuals aged under 12 years, hyperuricemia was defined as uric acid > 4.9 mg/dL. For males aged 12–19 years, it was uric acid > 7.6 mg/dL. For females aged 12–19 years, it was uric acid > 5.9 mg/dL.

The sample size determination was based on the prevalence (p) of dyslipidemia in cSLE, reported as 63% in a previous study by Tyrell et al. [35]. Using the formula n = Z^2^_ɑ/2_p(1-p)/*e*^2^, we calculated the minimum required sample size. The parameters used were type I error (α) = 0.05, allowable error (*e*) = 0.07 (12% of p), Z_ɑ/2_ = Z_0.025_ = 1.96, and p = 0.63. This calculation resulted in a minimum sample size of 182 participants. Accounting for a 10% dropout rate, the final sample size for this study was determined to be 200 participants.

All collected data were analyzed using IBM SPSS Statistics, version 28 (IBM Corp, Armonk, NY, USA). Continuous variables were tested for normality of distribution. Nonparametric variables were expressed as median and interquartile range (IQR). These were tested for significance using the Mann–Whitney U test. Parametric variables were expressed as mean ± standard deviation (SD) and tested for significance using independent sample t-tests. Categorical data were expressed as frequency and percentage. Analyses between categorical variables were conducted using the chi-square test or Fisher’s exact test. The relationships between variables were assessed using Pearson correlation coefficients (r) or Spearman correlation coefficients (rho), when appropriate. A *P* value of < 0.05 was considered significant. We adhered to the reporting guidelines described in the Strengthening the Reporting of Observational Studies in Epidemiology statement [36].

## Results

From a total of 171 cSLE patients, 39 were excluded for the following reasons: age under 10 years (*n* = 9), disease duration less than 12 months (*n* = 15), inability to communicate in Thai (*n* = 4), declined participation (*n* = 3), and dropout before assessment (*n* = 8). Therefore, the final number of participants was 132 cSLE patients.

The mean (± SD) age at cSLE diagnosis was 11.6 ± 2.6 years. The median age (IQR) at enrollment was 16.2 (14.1–17.8) years, and 125 patients (94.7%) were female. The total disease duration was 3.9 (2.0–5.8) years. The three most common cumulative organ involvements until study enrollment were hematological, mucocutaneous, and renal systems. At the time of assessment, the median (IQR) SLEDAI-2 K score was 2 (2–4), and the median (IQR) SLICC/ACR damage index was 0 (0–0). The LLDAS was achieved in 88 of 132 patients (66.7%). There were 23 of 132 cSLE patients with clinically active disease (17.4%), showing active features of organ involvement at assessment. The cumulative prednisolone equivalent dose was 11.3 (8.9–18.6) grams. Table 1 shows the clinical characteristics and laboratory results of the cSLE patients. Notably, two patients had been previously diagnosed with dyslipidemia, and two patients had developed diabetes mellitus prior to enrollment. However, none of them were on medications at the time of assessment or had the history of taking the medications for those diseases.

**Table 1:** Clinical characteristics and laboratory results of cSLE patients (*N* = 132)

Parameters	Value
**Demographic data**	
Age at diagnosis [years], mean (SD)	11.5 (2.6)
Age at assessment [years], median (IQR)	15.8 (13.8–17.6)
Duration of disease [years], median (IQR)	3.9 (2.0–5.8)
Sex: Female, n (%)	125 (94.7)
Body weight [kg], median (IQR)	49.7 (43.9–58.9)
Height [cm], median (IQR)	153 (148.1–157.2)
Waist circumference [cm], median (IQR)	73 (67–82)
Body mass index [kg/m^2^], median (IQR)	21.6 (18.5–25.1)
Body mass index z-score, median (IQR)	0.6 (0.4–1.5)
Hypertension, n (%)	37 (28.0)
Diabetes mellitus, n (%)	2 (1.5)
Dyslipidemia, n (%)	2 (1.5)
Family history of dyslipidemia, n (%)	14 (10.6)
Family history of metabolic syndrome, n (%)	1 (0.8)
Family history of diabetes mellitus, n (%)	12 (9.1)
Family history of cardiovascular disease, n (%)	4 (3.0)
Family history of obesity, n (%)	4 (3.0)
**SLE disease and cumulative organ involvement**	
Hematological, n (%)	110 (83.3)
Mucocutaneous, n (%)	93 (70.5)
Lupus nephritis, n (%)	66 (50)
Nephrotic syndrome, n (%)	28 (21.2)
Musculoskeletal, n (%)	48 (36.4)
Cardiovascular, n (%)	20 (15.2)
Pulmonary, n (%)	16 (12.1)
Gastrointestinal-hepatobiliary, n (%)	14 (10.6)
Neurological, n (%)	12 (9.1)
**SLE disease variables**	
ESR [mm/h], median (IQR)	14.0 (8.0–33.0)
UPCR, median (IQR)	0.1 (0–0.1)
Albumin [mg/dL], median (IQR)	4.3 (4.1–4.5)
Positive antinuclear antibody, n (%)	132 (100)
Positive anti-dsDNA, n (%)	90 (68.2)
Low C3 level, n (%)	47 (35.6)
Low C4 level, n (%)	51 (38.6)
SLEDAI-2 K at diagnosis, median (IQR)	13.0 (8.0–16.0)
SLEDAI-2 K at assessment, median (IQR)	2 (2–4)
SLICC Damage Index at assessment, median (IQR)	0 (0–0)
Lupus low disease activity state, n (%)	88 (66.7)
Clinically active disease at assessment, n (%)	23 (17.4)
**Current treatments**	
Cumulative prednisolone equivalent dose [g], median (IQR)	11.3 (8.9–18.6)
Prednisolone, n (%)	129 (97.7)
Hydroxychloroquine, n (%)	125 (94.7)
Azathioprine, n (%)	35 (26.5)
Mycophenolate mofetil, n (%)	32 (24.2)
Tacrolimus, n (%)	2 (1.5)
Antihypertensive medication, n (%)	36 (27.3)
**Metabolic laboratory variables**	
TC [mg/dL], median (IQR)	169 (144–198)
TG [mg/dL], median (IQR)	95 (75–144)
LDL-C [mg/dL], median (IQR)	103 (82–131)
HDL-C [mg/dL], median (IQR)	55 (46–66)
Fasting blood glucose [mg/dL], median (IQR)	79 (75–84)
HbA1C [mg%], median (IQR)	5.3 (5–5.5)
Uric acid [mg/dL], median (IQR)	5 (4–5.8)

Abbreviations: ESR: Erythrocyte Sedimentation Rate, HbA1C: Hemoglobin A1C, HDL-C: High-Density Lipoprotein Cholesterol, LDL-C: Low-Density Lipoprotein Cholesterol, SLEDAI-2 K: Systemic Lupus Erythematosus Disease Activity Index 2000, SLICC/ACR: Systemic Lupus International Collaborating Clinics/American College of Rheumatology, TC: Total Cholesterol, TG: Triglyceride, UPCR: Urine Protein-to-Creatinine Ratio

**Fig. 1:**
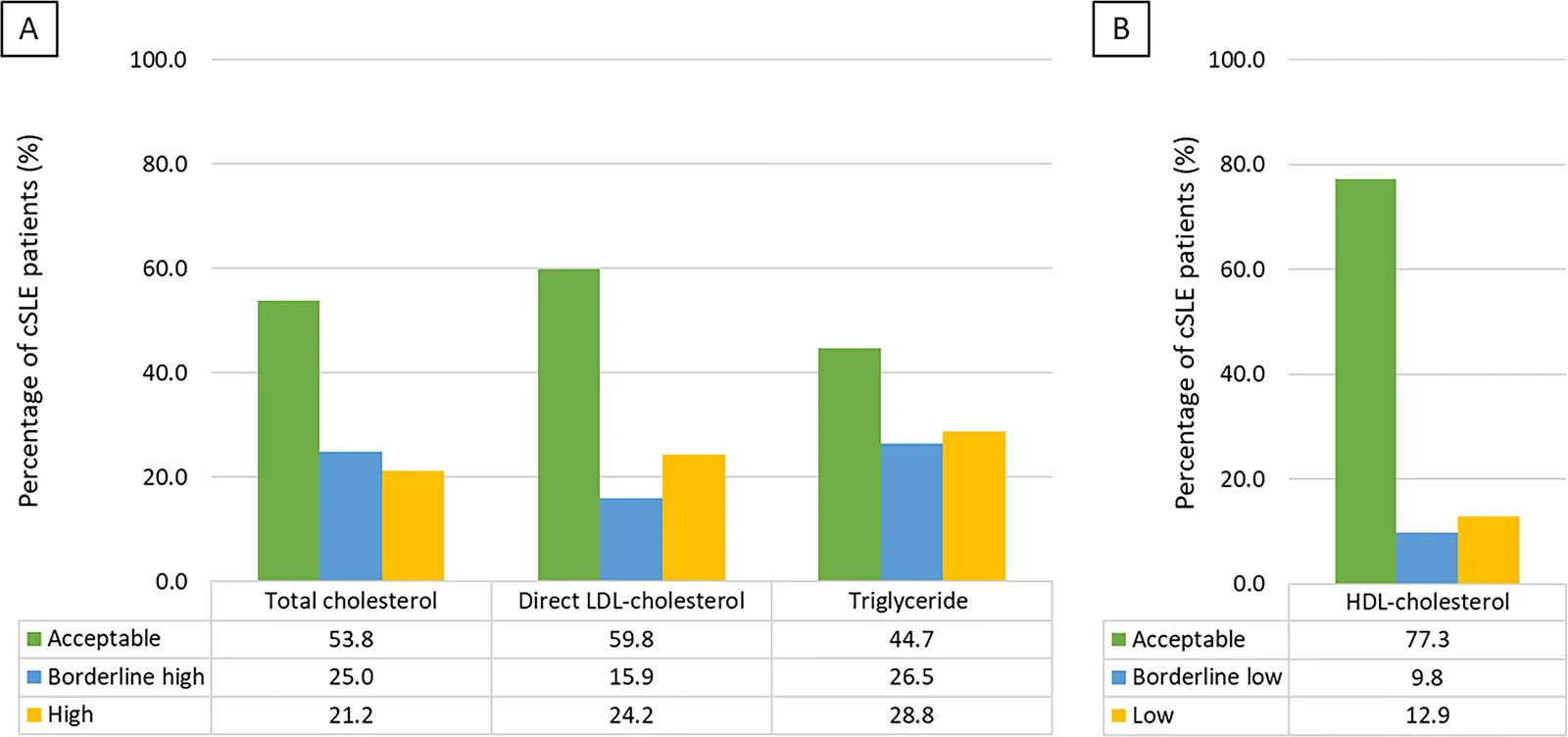
Percentage of cSLE patients in each stratified lipid profile category (*N* = 132). (**A**) Levels of total cholesterol (TC), low-density lipoprotein cholesterol (LDL-C), and triglycerides (TG) stratified as acceptable, borderline high, and high. (**B**) Levels of high-density lipoprotein cholesterol (HDL-C) stratified as acceptable, borderline low, and low

Dyslipidemia was prevalent in 64 patients (48.5%). Figure 1A and B detail the percentages of participants in each lipid category, as defined in the Methods. Hypertriglyceridemia was the most common lipid abnormality, occurring in 28.8% of all patients, followed by high direct LDL-C in 24.2% and high TC in 21.2% (Fig. 1A). Low HDL-C was the least common abnormality, occurring in 12.9% of patients (Fig. 1B). Metabolic syndrome was present in 3.8% of patients. Hyperuricemia was observed in 20.5%, and elevated FBG (≥ 100 mg/dL) was found in 0.8% of patients.

A comparison of clinical characteristics between cSLE patients with and without dyslipidemia revealed several statistically significant differences. The patients with dyslipidemia had younger age at diagnosis, higher percentage of patients with active features of organ involvement, higher percentage of hypertension, higher ESR levels, lower percentage of LLDAS, more frequent use of mycophenolate mofetil and antihypertensive medications, and higher uric acid levels (*P* < 0.05), as shown in Table 2. Additionally, cSLE patients with dyslipidemia had higher cumulative prednisolone equivalent dose than those without dyslipidemia; 12.8 (9.7–22.1) grams vs. 11.5 (8.9–16.5) grams, but did not achieve statistical significance (*P* = 0.064).

**Table 2:** Comparison of clinical characteristics and metabolic profiles between cSLE patients with and without dyslipidemia (*N* = 132)

Parameters	With dyslipidemia (n= 64)	Without dyslipidemia (n= 68)	*P* value
**Demographic data**			
Age at diagnosis [years], mean (SD)	11.1 (±2.4)	12 (±2.7)	0.043*
Age at assessment [years], median (IQR)	16.4 (14.9 − 17.6)	15.8 (13.5 − 17.9)	0.294
Duration of disease [years], median (IQR)	4.0 (2.0 − 6.2)	3.8 (2.0 − 5.3)	0.464
Sex: Female, n (%)	63 (98.4)	62 (91.2)	0.116
Hypertension, n (%)	25 (39)	12 (17.6)	0.006*
Underlying diabetes mellitus, n (%)	1 (1.6)	1 (1.5)	1.000
Underlying dyslipidemia, n (%)	2 (3.1)	0 (0)	0.233
**SLE disease variables**			
ESR [mm/h], median (IQR)	22 (10.3 − 43)	10.5 (8 − 19)	0.001*
UPCR, median (IQR)	0.1 (0 − 0.2)	0.1 (0.1 − 0.1)	0.130
Albumin [mg/dL], median (IQR)	4.2 (3.9 − 4.5)	4.3 (4.2 − 4.5)	0.088
Lupus nephritis class II	5 (7.8)	3 (4.5)	0.483
Lupus nephritis class III	8 (12.5)	4 (5.9)	0.186
Lupus nephritis class IV	14 (21.9)	16 (23.5)	0.821
Lupus nephritis class V	3 (4.7)	3 (4.4)	0.939
Lupus nephritis class III+V	0 (0)	3 (4.4)	0.245
Lupus nephritis class IV+V	3 (4.7)	4 (5.9)	0.759
Nephrotic syndrome	18 (26.5)	10 (15.6)	0.128
SLEDAI-2 K at assessment, median (IQR)	2 (2 − 4)	2 (2 − 4)	0.750
SLICC/ACR damage index at assessment, median (IQR)	0 (0−1)	0 (0−0)	0.109
Lupus low disease activity state, n (%)	37 (57.8)	51 (75.0)	0.036*
Clinically active disease at assessment, n (%)	16 (25.0)**	7 (10.3)***	0.026*
Systolic blood pressure [mmHg], median (IQR)	112 (106.3 − 121.8)	113 (105.3 − 117.8)	0.717
Diastolic blood pressure [mmHg], median (IQR)	67 (62.3 − 75.8)	67 (62.3 − 73)	0.547
**Current treatments**			
Hydroxychloroquine, n (%)	59 (92.2)	66 (97.1)	0.264
Prednisolone, n (%)	62 (96.9)	67 (98.5)	0.611
Daily prednisolone dose [mg], median (IQR)	5 (5−10)	5 (5-5)	0.056
Cumulative prednisolone equivalent dose [g], median (IQR)	12.8 (9.7−22.1)	11.5 (8.9−16.5)	0.064
Azathioprine, n (%)	19 (29.7)	16 (23.5)	0.423
Mycophenolate mofetil, n (%)	21 (32.8)	11 (16.2)	0.026*
Tacrolimus, n (%)	2 (3.1)	0 (0)	0.233
Antihypertensive medication, n (%)	25 (39)	12 (17.6)	0.006*
**Anthropometric variables**			
Body weight [kg], median (IQR)	53.2 (46.3 − 66.5)	49.7 (43.6 − 55.1)	0.020*
Height [cm], median (IQR)	152.3 (147.6 − 156.4)	154.8 (149.6 − 160)	0.036*
%Weight for age, median (IQR)	123.5 (95.2 − 144.3)	107 (93.9 − 113.9)	0.003*
%Height for age, median (IQR)	98 (95.4 − 100)	98.8 (95.9 − 101.6)	0.273
%Weight for height, median (IQR)	131.5 (106.7 − 160.8)	111.6 (99.4 − 127.8)	0.001*
Waist circumference, median (IQR)	77.5 (70.2 − 87.8)	71.3 (66.3 − 76.8)	0.001*
Body mass index, median (IQR)	23.4 (19.7 − 27.5)	20.3 (17.9 − 22.8)	<0.001*
Body mass index z-score, median (IQR)	1.01 (−0.48 − 1.84)	0.07 (−0.66 − 0.8)	<0.001*
**Metabolic laboratory variables**			
Fasting blood glucose [mg/dL], mean (SD)	80.4 (7.7)	79 (6.4)	0.266
HbAlc [mg%], median (IQR)	5.3 (5 − 5.6)	5.3 (5 − 5.4)	0.195
Uric acid [mg/dL], median (IQR)	5.2 (4.4 − 6.2)	4.5 (3.9 − 5.5)	0.018*
**Body composition**			
Fat mass [kg], median (IQR)	20.1 (14.5 − 28.7)	16.3 (12.3 − 21)	0.003*
Fat free mass [kg], median (IQR)	33.1 (29.6 − 36.8)	32.9 (29.1 − 36.3)	0.633
Skeletal mass [kg], median (IQR)	17.6 (15.2 − 19.6)	17.3 (14.9 − 19)	0.393
% Body fat, mean (SD)	38.3 (9)	32.9 (8.6)	0.001*
**Dietary consumption per day**			
Total calorie [kcal], median (IQR)	1050.7 (796.5 − 1325.2)	1157.4 (970.4 − 1370.9)	0.083
Protein [g], median (IQR)	43.4 (36.4 − 53.6)	47 (41.5 − 59.5)	0.060
Fat [g], median (IQR)	35.8 (26.9 − 48.7)	43.6 (36.1 − 55.4)	0.012*
Carbohydrate [g], mean (SD)	137.4 (55.6)	142.2 (47.9)	0.607

*Abbreviations* ESR: Erythrocyte Sedimentation Rate, HbA1c: Hemoglobin A1C, SLEDAI-2 K: Systemic Lupus Erythematosus Disease Activity Index 2000, SLICC/ACR: Systemic Lupus International Collaborating Clinics/American College of Rheumatology, UPCR: Urine Protein-To-Creatinine Ratio

*P < 0.05

**Organ involvement in patients with dyslipidemia: mucocutaneous in 7, renal in 5, hematological in 3, musculoskeletal in 1

***Organ involvement in patients without dyslipidemia: mucocutaneous in 2, renal in 2, hematological in 2, musculoskeletal in 1

**Table 3:** Correlations (r) between anthropometric measurements, lipid profiles, and disease status in cSLE patients (*N* = 132)

Variables	SLEDAI-2 K	PRED (mg/day)	ESR	UPCR	Albumin	BMI	WC	Fat Mass	TC	TG	HDL-C	LDL-C	Uric acid
SLEDAI- 2 K	1	0.341^**^	0.187	0.423^**^	− 0.421^**^	0.036	0.040	0.007	0.217	0.160	− 0.066	0.240^*^	0.113
PRED (mg/day)		1	0.327^**^	0.324^**^	0.259^*^	0.085	0.016	0.018	0.328^**^	0.297^*^	0.232^*^	0.166	− 0.033
ESR			1	0.273^*^	− 0.376^**^	0.204	0.180	0.167	0.248^*^	0.276^*^	− 0.128	0.329^**^	0.118
UPCR				1	− 0.609^**^	0.167	0.086	0.101	0.591^**^	0.472^**^	0.193	0.469^**^	0.109
Albumin					1	− 0.191	− 0.088	− 0.110	− 0.528^**^	− 0.372^**^	− 0.117	− 0.506^**^	− 0.281^*^
BMI						1	0.913^**^	0.971^**^	0.107	0.144	− 0.219	0.156	0.381^**^
WC							1	0.925^**^	0.063	0.122	− 0.279^*^	0.154	0.331^**^
Fat Mass								1	0.054	0.104	− 0.223	0.110	0.356^**^
TC									1	0.427^**^	0.429^**^	0.905^**^	− 0.005
TG										1	− 0.064	0.323^**^	0.224^*^
HDL-C											1	0.131	− 0.302^**^
LDL-C												1	0.057
Uric acid													1

*P < 0.01, **P < 0.001

*Abbreviations* BMI: Body Mass Index, ESR: Erythrocyte Sedimentation Rate, HDL-C: High-Density Lipoprotein Cholesterol, LDL-C: Low-Density Lipoprotein Cholesterol, PRED: Prednisolone; SLEDAI-2 K: Systemic Lupus Erythematosus Disease Activity Index 2000, TC: Total Cholesterol, TG: Triglyceride, UPCR: Urine Protein-To-Creatinine Ratio, WC: Waist Circumference

Concerning renal involvement, there were a total of 66 patients with lupus nephritis. Of this, there were 7 patients with active renal disease at the time of assessment; patients with dyslipidemia had higher frequency of active renal disease (5 of 64, 7.8%) than patients without dyslipidemia (2 of 68, 2.9%), although this did not reach statistical significance (*P* = 0.264). Proliferative lupus nephritis was found in 52 of 66 patients with lupus nephritis (78.8%); subgroup analysis between proliferative lupus nephritis and non-proliferative lupus nephritis did not show significant differences in the proportion of patients with dyslipidemia (25 of 52, 48.1% vs. 8 of 14, 57.1%; *P* = 0.547).

Anthropometric measurements showed that the dyslipidemia group had higher body weight, weight-for-age, weight-for-height, waist circumference, BMI, and BMI z-score than patients without dyslipidemia, with statistical significance (*P* < 0.05, Table 2). Additionally, body composition measured by bioelectric impedance analysis showed higher fat mass and fat percentage in the dyslipidemia group, with statistical significance (*P* < 0.05, Table 2). Detailed comparisons of clinical characteristics and metabolic profiles are presented in Table 2.

The relationships between anthropometry, lipid profiles, and SLE disease variables were explored. The correlation matrix of Pearson correlation coefficients (r) is presented in Table 3. Waist circumference, BMI, and body fat mass had significant positive correlations with uric acid levels (*P* < 0.001). The lipid profile variables TG, TC, and LDL-C showed significant positive correlations with UPCR (*P* < 0.001). TC exhibited significant correlations with daily prednisolone dose (*P* < 0.001). The lipid profile variables TG, TC, and LDL-C showed significant negative correlations with serum albumin (*P* < 0.001). HDL-C and uric acid were negatively correlated (*P* < 0.001). Spearman correlation was used for analyses of SLICC/ACR damage index and cumulative prednisolone equivalent dose with the variables of anthropometry, lipid profiles, and SLE disease. For SLICC/ACR damage index, which was considered to have damage if score > 1 and without damage if score 0, there was significant positive correlation with LDL-C (rho = 0.257, *P* < 0.01). Cumulative prednisolone equivalent dose showed significant positive correlations with TC (rho = 0.350, *P* < 0.001), LDL-C (rho = 0.351, *P* < 0.001), fat mass (rho = 0.227, *P* < 0.01), and UPCR (rho = 0.253, *P* < 0.01).

## Discussion

Our study explored the prevalence of dyslipidemia and metabolic syndrome in cSLE, the associations between lipid profiles, anthropometry, and disease status, and the factors correlating with the lipid profile levels.

The prevalence of dyslipidemia in our study (48.5%) aligns with previous reports, which have documented rates ranging from 39.4 to 67.7% in cSLE patients [35, 37–39]. The variations in the percentage between studies may be attributed to differences in study designs and timing of assessments during the disease course. We observed hypertriglyceridemia as the most common lipid abnormality in nearly 30%, while low HDL-C was the least common. Prior studies have reported patterns of decreased HDL-C and increased TG, TC, and LDL-C, especially in active cases where hypertriglyceridemia and low HDL-C are commonly observed [35, 38–40]. However, the underlying mechanisms explaining these lipid profile patterns in cSLE patients remain unclear.

Our study identified several factors associated with dyslipidemia in cSLE patients. Patients with dyslipidemia had younger age at SLE diagnosis and higher percentage of hypertension compared to those without dyslipidemia. They also exhibited higher percentage of active features of organ involvement, higher ESR levels, lower percentage of LLDAS, and were more likely to be currently receiving mycophenolate mofetil. Although the pathogenesis of dyslipidemia in SLE is not well-established, previous studies have revealed that abnormal lipid profiles are related to inflammation and abnormal immunity. Treatments including corticosteroids, cyclosporin, and tacrolimus have also been implicated in the development of dyslipidemia [39–45]. Previous studies have found that lipid abnormalities—notably hypertriglyceridemia, hypercholesterolemia, and elevated LDL-C—are associated with renal involvement, proteinuria, and increased SLE activity [35, 40, 46, 47]. Studies in cSLE patients with nephritis reported higher levels of TC, TG, and LDL-C than those without nephritis [35, 46]. Positive correlations between nephritis and TG, and negative correlations between nephritis and HDL-C have been reported [35]. Dysregulated lipid profiles have been reported to have associations with the SLEDAI-2 K in cSLE [37, 47]. From our results, the dyslipidemia group had slightly higher percentage of active renal disease at assessment. There were no differences in the lupus nephritis classes between cSLE patients with and without dyslipidemia, or the differences in the frequency of dyslipidemia between patients with proliferative lupus nephritis and non-proliferative lupus nephritis. These findings may have been influenced by factors such as physical activity, pubertal onset, and pubertal status, which were not assessed in our study. Moreover, our study protocol evaluated cSLE patients at least 1 year after diagnosis and treatment for SLE, resulting in the majority of patients having inactive disease. Notably, most patients were on hydroxychloroquine, which benefits lipid profiles [7]. Regarding assessment of ESR, hyperlipidemia can cause elevation of ESR, and because of the complex relationship between cSLE disease activity, treatment, and lipid profiles [47], it would be difficult to determine whether elevated ESR was attributed to hyperlipidemia or active disease itself.

In our study, correlation analysis showed that the UPCR positively correlated with TG, TC, and LDL-C. This aligned with the studies in cSLE which showed the significant associations of dyslipidemia and proteinuria [37, 47]. Serum albumin also had negative correlations with TG, TC, and LDL-C. These findings were consistent with studies that showed abnormal lipid profiles were associated with serum albumin levels [35, 47–49]. Dyslipidemia in patients with proteinuria could be explained by altered lipid metabolism due to impaired lipoprotein lipase activity, decreased hepatic lipase activity and the presence of anti-lipoprotein lipase antibodies [7, 37, 50]. Moreover, our study supports the effect of treatment with corticosteroids which potentially impact lipid metabolism, both long-term as presented by cumulative prednisolone equivalent dose, and cross-sectionally as presented by daily prednisolone dose. TC and LDL-C positively correlated with cumulative prednisolone equivalent dose, and TC positively correlated with daily prednisolone dose. From previous studies, abnormal lipid profiles in cSLE can be attributed to the treatment effects of systemic corticosteroids [7, 39–41]. We highlight the clinical implications for cSLE patients with higher UPCR and higher cumulative prednisolone equivalent dose to be screened earlier and regularly for abnormal lipid profiles.

Anthropometric analysis from our study showed that the dyslipidemia group had higher BMI and BMI z-scores. Body composition analysis revealed higher fat mass in this group but lower fat intake. These controversial findings could be explained by the possibility that cSLE patients with higher BMI had received dietary counseling during routine follow-ups before enrollment.

Metabolic syndrome was detected in 3.8% of our cSLE patients according to the International Diabetes Federation criteria [17]. This is similar to the data observed in Thai children in 4% [51]. From a global perspective, metabolic syndrome was prevalent in 3% of children and 5% of adolescents, with some variations across countries and regions [52] e.g. 2.8% of children and 7.8% of adolescents in the USA, 1.4% of children and 3.7% of adolescents in northwestern Europe, 5.6% of children and 5.4% of adolescents in Latin America and Caribbean, 3.5% of children and 5.6% of adolescents in the Middle East and north Africa, and 2.1% of children and 4.8% of adolescents in Southeast Asia [52]. The prevalence of metabolic syndrome in cSLE from our study was comparable to normal population, and could be implied that cSLE patients may not have increased risk of metabolic syndrome. Nevertheless, the criteria for diagnosing metabolic syndrome in pediatric populations vary, which could lead to differences in reported prevalence rates [19, 53, 54]. Interestingly, we found the discrepancy between the prevalence of metabolic syndrome and dyslipidemia in cSLE patients. This could possibility be due to the definition for metabolic syndrome requiring fulfilment of the criteria assessing waist circumference, fasting TG, fasting HDL-C, blood pressure, and FBG, thus some patients may have not met the criteria, whereas the definition for dyslipidemia in our study was the presence of at least one abnormal lipid profile (high TC, LDL-C, TG, or low HDL-C). Another possible explanation could be that the criteria proposed by the International Diabetes Federation has yielded lower prevalence of metabolic syndrome among children and adolescents compared to other criteria [54]. Studies on long-term follow-up of cSLE patients with metabolic syndrome and the development of premature atherosclerosis transitioning into adulthood are still scarce. For clinical implications, we emphasize early detection of metabolic syndrome in cSLE patients age 10 years or older and also in those younger than 10 years who are obese or overweight with features of metabolic syndrome or are potentially at risk of having metabolic syndrome, by applying the proposed definition [17]. Nevertheless, there is a need to develop a consistent criteria in the pediatric population for early identification of patients at risk [53, 54].

Our study revealed hyperuricemia in one-fifth of cSLE patients. Uric acid levels showed positive correlations with BMI, waist circumference, and fat mass but a negative correlation with HDL-C levels. Hyperuricemia has been considered as a clinically relevant cardiometabolic risk, but has not been included in the criteria for metabolic syndrome in children and adolescents [54]. In children with hyperuricemia, dyslipidemia, and metabolic syndrome, there should be awareness of the potential to develop juvenile gout, even though it is rare in this age group. However, gout has rarely been reported in cSLE; one study which explored musculoskeletal manifestations in cSLE at initial diagnosis and complications during the disease course did not identify any cases of gout [55]. Nevertheless, studies of the clinical associations and pathogenesis of hyperuricemia in cSLE are scarce. For clinical implications, we encourage that screening uric acid levels in cSLE patients can be beneficial, as hyperuricemia is associated with parameters related to dyslipidemia, metabolic syndrome, and SLE disease activity.

Several limitations of this study should be acknowledged. First, certain unmeasured factors and incomplete data may have influenced our findings. The lack of assessment for physical activity and pubertal status could have affected the lipid profiles. Additionally, some patients had missing data in their food record forms, which may have impacted the calculation of daily caloric intake. Second, the cross-sectional study design measured variables at a single time point, making it challenging to infer causality, without a control group of healthy participants. Anthropometry, body composition, and lipid profiles can vary over time and may be influenced by disease status, treatment, and dietary intake. Therefore, future longitudinal studies for multiple assessments over time could provide more accurate insights, with additional assessments of physical activity factors, pubertal status and menarche, giving more comprehensive understanding of dynamic changes in lipid and metabolic profiles. Such studies could shed light on the underlying causes of abnormalities. Third, this study was performed at a single-referral center and thus may not reflect all cSLE patients in the general population. Nevertheless, it assessed cSLE patients who had been diagnosed and treated for at least 1 year at the nation’s largest tertiary referral center. This setting could represent real-life practice based on standard patient care recommendations, with additional exploration of metabolic factors related to SLE. Patient and parent education on cardiovascular risk factors and lipid profile screening should be provided at regular intervals [20]. Although the need for treatment of dyslipidemia and/or metabolic syndrome in cSLE patients was beyond the context of this study, in practice all cSLE patients with identified dyslipidemia and/or metabolic syndrome received counselling for lifestyle modification regarding diet control, adequate physical activity, education for cardiovascular risks, follow-up of blood tests and anthropometric measurements, and initiation of lipid lowering drugs, when indicated.

## Conclusions

Dyslipidemia is common in cSLE patients and is associated with SLE disease activity, metabolic comorbidities, anthropometry, and body composition. Additionally, metabolic syndrome and hyperuricemia can be present in these patients. Based on our findings, we encourage the screening and regular monitoring of lipid profiles, uric acid levels, and anthropometric measurements in cSLE patients at the younger age, particularly for patients aged 10 years or older with higher BMI, higher cumulative dose of systemic corticosteroids, proteinuria, and suboptimal-controlled SLE. Early detection of at-risk patients can facilitate proper management and improve outcomes for cSLE patients.

## Data Availability

Data are available from the corresponding author upon reasonable request.

## Abbreviations

ACR: American College of Rheumatology
BMI: Body Mass Index
cSLE: Childhood-onset Systemic Lupus Erythematosus
ESR: Erythrocyte Sedimentation Rate
FBG: Fasting Blood Glucose
HbA1c: Hemoglobin A1c
HLD-C: High-Density Lipoprotein Cholesterol
IQR: Interquartile Range
INMUCAL: Institute of Nutrition, Mahidol University Calculation *(Nutritional Analysis Program)*
LDL-C: Low-Density Lipoprotein Cholesterol
LLDAS: Lupus Low Disease Activity State
NCEP-ATPIII: Third Report of the National Cholesterol Education Program Expert Panel on Detection, Evaluation, and Treatment of High Blood Cholesterol in Adults
SD: Standard Deviation
SLE: Systemic Lupus Erythematosus
SLEDAI-2 K: Systemic Lupus Erythematosus Disease Activity Index 2000
SLICC: Systemic Lupus International Collaborating Clinics
TC: Total Cholesterol
TG: Triglycerides
UPCR: Urine Protein-To-Creatinine Ratio
