# Correction: Dyslipidemia and metabolic syndrome in childhood-onset systemic lupus erythematosus: is it time to screen?

**DOI:** 10.1186/s12944-026-03061-7

**Published:** 2026-09-29

**Authors:** Sirin Nuntasri, Sirirat Charuvanij, Kraisoon Lomjansook, Puthita Saengpanit, Kwanjai Chotipanang, Maynart Sukharomana

**Affiliations:** 1 Division of Rheumatology, Department of Pediatrics, Faculty of Medicine Siriraj Hospital, Mahidol University, 2 Wanglang Road, Bangkoknoi, Bangkok, 10700 Thailand; 2 Division of Nephrology, Department of Pediatrics, Faculty of Medicine Siriraj Hospital, Mahidol University, Bangkok, Thailand; 3 Division of Nutrition, Department of Pediatrics, Faculty of Medicine Siriraj Hospital, Mahidol University, Bangkok, Thailand

**Correction: Lipids Health Dis 23, 406 (2024)**

**https://doi.org/10.1186/s12944-024-02395-4**

Following publication of the original article [1], the authors identified an error in the two Table 1 variables.

**Table:** 

Body mass index z-score, median (IQR)	0.4 (0.6–1.5)
TC [mg/dL], median (IQR)	144 (169–198)

The two variables should read:

**Table:** 

Body mass index z-score, median (IQR)	0.6 (0.4–1.5)
TC [mg/dL], median (IQR)	169 (144–198)

The original article has been updated.
